# An Exploratory Study Investigating Factors Influencing the Outpatient Delivery of Geriatric Rehabilitation

**DOI:** 10.3390/jcm12155045

**Published:** 2023-07-31

**Authors:** Lidy A. P. Prins, Chris J. Gamble, Eléonore F. van Dam van Isselt, Romy A. I. Stammen, Ahlam Ettaibi, Ilse A. M. Creemers, Jolanda C. M. van Haastregt

**Affiliations:** 1 Department of Health Services Research, Faculty of Health Medicine and Life Sciences, CAPHRI Care and Public Health Research Institute, Maastricht University, 6229 ER Maastricht, The Netherlands; lidy.prins@maastrichtuniversity.nl (L.A.P.P.); c.gamble@maastrichtuniversity.nl (C.J.G.); 2 Stichting Valkenhof, 5555 KL Valkenswaard, The Netherlands; 3 Department of Public Health and Primary Care, Leiden University Medical Centre, 2333 ZA Leiden, The Netherlands; e.f.vandamvanisselt@lumc.nl; 4 Faculty of Health Medicine and Life Sciences, Maastricht University, 6229 ER Maastricht, The Netherlands; r.stammen@alumni.maastrichtuniversity.nl (R.A.I.S.); a.ettaibi@alumni.maastrichtuniversity.nl (A.E.); i.creemers@alumni.maastrichtuniversity.nl (I.A.M.C.)

**Keywords:** geriatric rehabilitation, outpatient, home-based, facility-based, frail older people, barriers, case study

## Abstract

Background: Outpatient delivery of geriatric rehabilitation (GR) might contribute to preserving the accessibility and quality of GR, whilst dealing with an increasing demand for healthcare in an aging population. However, the application of outpatient GR differs between GR facilities. This study aimed to gain insight into factors influencing outpatient GR utilization. Methods: In this case study, 24 semi-structured interviews were conducted with physicians, physiotherapists, nurse practitioners, occupational therapists, and managers in GR. Interviews were transcribed and analyzed using summative content analysis. Results: Various patient-related barriers for using outpatient GR were mentioned including lacking social support and limited capacities and self-management skills. Additionally, professional-related barriers included a lack of awareness and consensus among care professionals regarding the possibilities and potential advantages of outpatient GR. Yet, most perceived barriers were related to efficiency and organization of outpatient GR (e.g., reimbursement system, lacking practical guidance). Still, most participants were in favor of increasing outpatient GR because of expected advantages for patients, GR organizations, and society. Conclusions: Despite experienced barriers, there seems to be agreement on the need to increase outpatient GR application. It is recommended to use the present findings to develop and evaluate new ways of organizing and reimbursing outpatient GR.

## 1. Introduction

Geriatric rehabilitation (GR) is a multidimensional approach of diagnostic and therapeutic interventions that aim to optimize functional capacity, promote activity, and preserve both functional reserve and social participation in frail, older people who have disabling impairments [1,2]. Specifically, GR is aimed at community-living persons with multimorbidity and geriatric syndromes, who still have improving potential in their functional performance [2]. In the Netherlands, GR is primarily indicated after hospital admission, but in the case of an acute condition resulting in mobility impairment and/or loss of independence, it is also possible to be referred to GR by the general practitioner directly from home [3].

Dutch GR is provided by a multidisciplinary team led by a physician specialized in the care for older persons. Dependent on the rehabilitation aims, several other disciplines including physiotherapists, occupational therapists, dietitians, speech therapists, and psychologists are involved [3]. Typically, in the Netherlands, the multidisciplinary GR team works at a GR facility which is often situated near (or in) a nursing home or hospital. The GR team works in a multi- or an interdisciplinary way in which disciplines have overlapping expertise and clinical focus, yet maintain a discipline-specific base reserving exclusive competencies [4,5]. Teams follow an integrated approach by developing collective treatment plans to achieve the rehabilitation goals of the patient [4]. GR teams distinguish themselves from primary care professionals based on their multi- or interdisciplinary approach. Patients only receive GR when they have multidisciplinary rehabilitation goals; in the case of monodisciplinary rehabilitation goals, they are treated by primary care professionals [6].

In a meta-analysis, Bachmann and colleagues showed that GR improves function and reduces nursing home admission and mortality [7]. Despite these favorable outcomes, several developments complicate the quality and accessibility of GR as presently organized. Most notably, it is expected that the aging of the population will increase the prevalence of chronic diseases and multimorbidity among older persons, likely increasing the demand for GR and increasing the pressure on an already strained healthcare labor market [8,9,10,11,12,13,14]. Consequently, the resulting unmet care demand could eventually lead to increased healthcare problems and increased healthcare costs [15,16].

Because of these challenges, new strategies must be developed to preserve the quality and accessibility of GR. Increasingly, there is a preference among experts for the outpatient organization of GR [17], which might contribute to solving these challenges. Outpatient GR entails a similar delivery to inpatient GR delivered by a multidisciplinary GR team; however, it is applied either home-based or facility-based [17,18,19] and aims for optimal functioning of the patient in the home environment. Outpatient GR is expected to both enhance independent living and functional abilities [20] and to deliver more person-centered and improved care, more satisfied patients, and lower costs for society [21]. Over the past few decades, research on the effectiveness, cost-effectiveness, and feasibility of outpatient GR has been scarce [17,18,22]. Recently, Preitschopf and colleagues conducted a systematic review and meta-analysis and showed equal effectiveness for functional performance of outpatient GR compared to usual care [19]. Additionally, this review included eight studies that reported on cost-effectiveness, of which six showed a beneficial effect of outpatient GR [19].

Nonetheless, the application and organization of outpatient GR differ between GR facilities and countries [19,23], and outpatient GR is only used to a limited extent in the Netherlands [18]. Initial insights in the Dutch regulatory context illuminate potential barriers for the feasibility of outpatient GR that might help to explain the limited application. Examples include practical matters (e.g., travel time and transport, practitioners’ full schedules, patient’s physical capacity), complex regulations of outpatient GR, and the reimbursement structure making it hard to reach a break-even point in the costs and benefits of outpatient GR (e.g., lacking travel allowance for the care professional) [18,21,22,24,25].

Nevertheless, van den Besselaar and colleagues indicated in a study that the providers of GR want to increase the utilization of outpatient GR. However, in accordance with the aforementioned barriers, they also concluded that it seems challenging to accomplish this [22]. Furthermore, a study by van den Bosch and colleagues revealed that care providers and experts in the domain of GR expect that a larger part of the current inpatient GR can be provided as outpatient GR [18]. It seems that care professionals experience a sense of urgency to expand outpatient GR. To further assess the potential of outpatient GR, additional insight into views regarding outpatient GR of care professionals is necessary. Therefore, this study aimed to gain insight into the opinions of care professionals employed in Dutch GR facilities on factors influencing the use and further increase in outpatient GR trajectories.

## 2. Methods

### 2.1. Design

The present study has an explorative, qualitative multiple case study design, which allows for rich data collection and facilitates an in-depth exploration of the views of GR professionals on factors influencing the delivery of outpatient GR [26].

### 2.2. Population and Setting

This study included the main disciplines in the GR team, consisting of physicians specialized in the care for older persons, nurse practitioners, physiotherapists, and occupational therapists. Furthermore, GR managers were included since they play a crucial role in the decision-making process regarding outpatient GR and were recruited at different executive levels in the GR organizations (i.e., facility-level and team-/department-level). Participants were recruited until data saturation was reached. Data saturation relates to the degree to which new data repeat what was expressed in previous data, in other words, a point where there appears diminishing results from further data collection. This is based on the researchers sense of what they are hearing during the interviews [27]. Interviewing these different disciplines ensured exploration of different perspectives on factors influencing the delivery of outpatient GR. Recruitment followed the purposive sampling principle [28] and was executed by a senior researcher (JvH) of Maastricht University, with a professional network in the domain of GR. Participants were either selected directly or via contact persons in GR facilities. To be eligible for inclusion, the GR professional had to be employed in a Dutch GR facility for at least half a year, needed to have knowledge on the status of outpatient GR in their GR facility, and had to provide informed consent for participation.

### 2.3. Data Collection

Data were collected through semi-structured interviews based on a topic list to ensure certain topics were covered in all interviews. Topics included in the interview were the current use of outpatient GR in the facility where the care professional is employed, factors influencing the feasibility of outpatient GR delivery, opinions on increasing the proportion of outpatient GR trajectories, and perceived prerequisites to enable the increase in outpatient GR trajectories. In addition, participant characteristics were collected at the start of the interview (i.e., sex, age, and years of experience in the current GR facility). Interviews were, based on the participants’ preferences, conducted either face-to-face or online using video call software. With the permission of the participants, interviews were recorded.

### 2.4. Data Analysis

The interviews were transcribed and analyzed by the researchers using ATLAS.ti software guided by the summative content analysis approach [29]. This implied that, before data analysis, keywords were identified (based on the main themes on the topic list) that constituted the foundation for analysis and ensured consistency between the researchers. Additionally, in this approach during data analysis, additional codes can emerge (i.e., manifest content). This enabled exploring factors inductively whilst also working with a predetermined focus. The researchers (LP, RS, AE, and IC) analyzed different discipline subgroups. During these analyses, the researchers evaluated preliminary findings with the coordinating senior researcher (JvH) who monitored the consistency of the analysis. First, the researchers intensively read the transcribed interviews to become immersed in the data. Hereafter, the coding scheme was expanded; prominent themes and patterns were identified by breaking down data into smaller units; and labels were attached (i.e., codes) [29]. Based on this analysis, written summaries of the interviews were sent to the participants to execute a member check [30] that had to verify whether the researchers’ interpretations correctly expressed the participants’ input. The researchers processed the resulting participant feedback in the analysis. Finally, one researcher (LP) integrated the findings of the researchers in collaboration with the senior researcher (JvH).

## 3. Results

### 3.1. Participants

Twenty-four care professionals from five different GR disciplines (Table 1), working at nineteen different GR facilities participated in the study. The largest subgroup involved physicians specialized in the care for older persons. A total of 3 participants were male, 21 participants were female, and their ages ranged from 25 to 63 years.

### 3.2. Current Delivery of Outpatient GR

Currently in the Netherlands, outpatient GR is only reimbursed when it is preceded by inpatient GR. Participants were asked to estimate the percentage of GR trajectories that include outpatient GR delivery. The estimated percentages ranged from 0 to 60%, in which estimations between 1 and 10% were most frequently reported (eleven times), followed by seven estimations of 0%, four estimations between 10 and 25%, one estimation between 25 and 40%, and one estimation between 40 and 60%.

### 3.3. Factors Influencing the Delivery of Outpatient GR

Indicated factors influencing the delivery of outpatient GR were categorized into patient-related factors, care-professional-related factors, and factors related to the efficiency and organization of outpatient GR.

#### 3.3.1. Patient-Related Factors

Frequently indicated patient-related factors hindering outpatient GR were the absence of a sufficient social support system, impaired capacities or fitness of the patient (both physical and cognitive), and limited self-management skills (Quote 1). Moreover, in facility-based GR, the patient’s social support system often plays a crucial role in organizing transport possibilities to the GR facility (Quote 2), potentially determining the outpatient options. Additionally, it was indicated that occasionally patients do not want an outpatient GR trajectory, for example, because they were insecure about whether they were already able to leave the facility.

Quote 1: *“If someone needs a bit of support, but does not have a partner, then it’s complicated to set up an outpatient program at all, because someone must have a certain level of self-management before you can make that step home at all. But if you have someone around you who can provide some support, then you are ready to go home sooner than when you live alone”*

—(Physician).

Quote 2: *“[…] patients often depend on others to come to us, at least most of them in our target group, so that makes it more difficult for the patient.”*

—(Physiotherapist).

#### 3.3.2. Care-Professional-Related Factors

A factor indicated as a barrier to outpatient GR was a lack of awareness about the possibilities and potential advantages of outpatient GR among the GR professionals. Closely related to this, it was indicated that lacking consensus about the necessity to provide outpatient GR can hinder the delivery of outpatient GR. Several GR professionals also indicated that it is difficult to assess at what moment the inpatient GR trajectory can be continued outpatient. Additionally, it was regarded as a risk by some GR professionals that when patients receive outpatient GR, there is no constant monitoring and control (Quote 3), which for some might result in being somewhat reluctant to continue in an outpatient trajectory.

Quote 3: *“[…] if there is a patient who […] has broken a hip, and needs to go to the toilet at night, for example… Yes, if someone is home alone, I find that rather worrying, so to speak. You just have to hope that it goes well.”*

—(Physiotherapist).

#### 3.3.3. Factors Influencing Efficiency of Outpatient GR

The vast majority of the GR professionals indicated barriers related to the reimbursement regulations that currently apply to outpatient GR in the Netherlands. Several examples were provided including hindering reimbursement requirements and specifically the lack of reimbursement of travel time and costs in cases of home-based GR. Regarding these reimbursement-related barriers, multiple GR professionals indicated subsequently that GR facilities currently still experience difficulties to provide outpatient GR cost-effectively. Regardless of the lacking reimbursement of travel time and costs, travel time and distance were indicated as hindering factors. In cases of home-based GR, this affects the GR professional’s time (Quote 4), also since there is an increased social role reserved for the care professional as more time is spent on “socializing” (Quote 5). In cases of facility-based GR, this applies to the patient (Quotes 2 and 6).

Quote 4: *“During the time we went to visit the patient at home, we would be able to treat double the number of patients at the geriatric rehabilitation center.”*

—(Physician).

Quote 5: *“[…] you have a chat, people want to show you things […]”*

—(Physiotherapist).

Quote 6: *“Transport from A to B is always tricky. Can someone be brought, or should he take a taxi? Does he have to take a group taxi, which has to drive a route with 10 people, which is again extra stressful”*

—(Physician).

#### 3.3.4. Factors Related to Organization of Outpatient GR

Regarding the content and application of both home-based and facility-based GR, the lack of adequate exercise and treatment facilities for patients receiving outpatient GR was indicated as a hindering factor by several GR professionals (e.g., lack of exercise materials at home or waiting rooms at the GR facility). Furthermore, the staff shortage was considered as a barrier to outpatient GR. Some participants explained additionally that the outpatient trajectories increase the total number of GR patients since often the vacant beds are directly reoccupied by new inpatients which increases the workload (Quote 7). Besides, the number of outpatient trajectories varies per period. Both factors complicate arranging the required staffing levels adequately. Additionally, it was indicated that home care possibilities are also scarce (Quote 8), due to staff shortage in community care. This complicates the outpatient GR options as the availability of home care is, for part of the GR population, a precondition for inpatient discharge. Furthermore, several participants indicated a lack of clear guidance on the content of outpatient GR. Consequently, for them, this hinders the application of outpatient GR.

Quote 7: *“What I was wondering is whether we are going to manage this with the current staff, because look, as soon as a bed becomes unoccupied, this man continues with an outpatient GR trajectory, however, this bed is immediately occupied again, so then you actually have two patients for one bed.”*

—(Physiotherapist).

Quote 8: *“[…] currently notice that people sometimes have to wait on the rehabilitation ward until home care has been arranged”*

—(Physiotherapist).

### 3.4. Opinions on Increasing the Proportion of Outpatient Trajectories

The large majority was in favor of an increase in outpatient trajectories. Several arguments were indicated for this, related to the added value for the patient, GR organization, and society.

The most frequently indicated arguments were related to GR professionals’ judgment that patients recover best in their home environment. This environment offers the care professional the ability to observe the patient in executing activities of daily living (Quote 9) and it offers opportunities to functionally exercise with the patient. Related to this, some participants indicated that outpatient GR could enhance peoples’ ability to remain living, independently at home for a longer time, for instance, by reinforcing patients’ support systems in the outpatient trajectory. Furthermore, it was indicated that functioning in one’s surroundings triggers doing activities independently and that therefore it might enhance independence. Whilst, it was also indicated that at home, informal caregivers sometimes take over too many tasks and therefore create a risk of dependability. Additionally, some participants indicated that outpatient GR might ensure a smoother transition toward the home environment (Quotes 10 and 11). In part, this is because outpatient GR provides the opportunity to continue the involvement of the same GR professionals, although, in practice, this is not always perceived feasible (Quote 7). Furthermore, participants indicated that outpatient GR might reduce pressure on the clinical beds and that direct outpatient GR (i.e., without preceding clinical admission) should be an option as well. Related to this, several participants estimated that outpatient GR might reduce costs compared to the present GR organization that primarily follows a clinical setup (Quote 12).

Regarding the organization of facility-based GR, several participants indicated that the option to offer facility-based group therapy can have several advantages for the patient and the GR organization. For the GR organization, this entails more efficient care planning as multiple (clinical and outpatient) patients can be treated simultaneously. For the patient, this entails a motivating effect, decreased loneliness, and the opportunity to utilize the organization’s facilities. Yet, regarding the eventual content of outpatient GR, several participants indicated that a combination of facility-based GR and home-based GR should be possible since patients should be able to receive a tailor-made treatment.

In contrast, a small minority of GR professionals was against an increase in outpatient trajectories, for instance, arguing that outpatient GR is merely of added value for a small part of the GR population. Hence, it was considered questionable whether the limited added value for only a small population is sufficient to increase outpatient GR trajectories. Additionally, it was indicated that it should be assessed for which specific target group outpatient GR is especially of added value. Moreover, it was perceived that outpatient rehabilitation can also be offered by care professionals in primary care. Yet, according to almost all participants, the added value of outpatient GR compared to primary care is the multidisciplinary nature of outpatient GR. Additionally, it was indicated that considering the complexity of certain GR cases, the expertise in outpatient GR is also an important added value.

Quote 9: *“Maybe when you see someone functioning in their own environment, you see the total picture, that you see someone going over thresholds in their home or in a shower with a 30 cm step”*

—(GR manager).

Quote 10: *“[…] from having here 24/7 staff around, to being completely home alone again […]. We simply want that transition to be a little smoother […]”*

—(Physiotherapist).

Quote 11: *“If you visit someone for 2–3 weeks, they have that, that confidence of: “okay, I can do this at home””*

—(Physiotherapist).

Quote 12: *“[…] beds in an institution are just really expensive and a bed at home is not that expensive”*

—(Occupational therapist).

### 3.5. Prerequisites for Increasing the Proportion of Outpatient Trajectories

The main perceived prerequisites to enable an increase in the proportion of outpatient GR trajectories were related to the efficiency and organization of outpatient GR, the care professionals involved, and the patients. Regarding efficiency of outpatient GR, a suitable reimbursement system is needed. Examples were reorganizing the reimbursement requirements and the reimbursement of travel costs and time to increase the outpatient GR possibilities. Furthermore, development of guidance for outpatient GR is a perceived prerequisite (e.g., care pathways or guidelines) (Quote 13). Additionally, increased involvement of health insurers is perceived necessary to further develop outpatient GR. For instance, this can be realized by facilitating manageable information about reimbursement, organization and application, eligible patients, and advantages of outpatient GR. Furthermore, health insurers could arrange opportunities to pilot eHealth applications and technology in outpatient GR. It was indicated that it is currently difficult to explore the use of technology due to its limited reimbursement. Finally, a sufficiently large and flexible workforce is perceived as a prerequisite.

Regarding the care professionals involved, good communication and collaboration (including a proper multidisciplinary consultation setup) are perceived needed between all care professionals involved, including primary care professionals such as community nurses, general practitioners, or their practice assistants (Quote 14). Additionally, a positive intention of the organization and its employees regarding outpatient GR needs to be established (Quote 15). The major patient-related prerequisites to increase the proportion of outpatient trajectories were sufficient physical capacity of the patient and sufficient support at home, both material and immaterial. Moreover, for facility-based GR, there must be no unresolvable transport issues present for the patient.

Quote 13: *“If the frameworks were much clearer and more streamlined, I think that would help everyone, because now everyone is inventing their wheel, each wheel looks slightly different. That has to do with regional differences, willingness of people in the region, whether they want to invest in themselves? How are things already arranged? Or you can link up with certain structures that already exist, but it is all a bit improvised, because funding and organization are not properly framed”*

—(Physician).

Quote 14: *“For example, home care does a piece of care, the therapist who visits for a walk, there may also be the physician’s practice assistant on the team because there is a psychosocial need. How do you connect those people that they communicate with each other for the sake of that client?”*

—(GR manager).

Quote 15: *“Yes, I do think that you must have the organization or the management with you, because ultimately, if you want to expand outpatient then the hours must expand as well. And if the organization says, we won’t do that then, that’s a limiting factor”*

—(Occupational therapist).

## 4. Discussion

This study aimed to gain insight into the opinions of care professionals employed in Dutch GR facilities on the current use of outpatient GR and on factors influencing its use. Of all participants, seven reported no current delivery of outpatient GR in their facility, and seventeen reported using outpatient GR only to a limited extent.

Factors influencing the feasibility of outpatient GR were categorized into factors related to patients, care professionals, and the efficiency and organization of outpatient GR. Important patient-related barriers for outpatient GR were the absence of a sufficient social support system, impaired capacities or fitness of the patient (both physical and cognitive), and patients who do not want an outpatient trajectory. The main care-professional-related barriers were a lack of awareness and consensus about the possibilities and potential advantages of outpatient GR. Furthermore, it appears to be difficult for the care professionals to judge when to continue with outpatient GR. Most perceived barriers were related to the efficiency and organization of outpatient GR and often related to reimbursement regulations, such as lacking reimbursement of travel time and costs, which seem to complicate a cost-effective and feasible outpatient GR. Furthermore, other barriers related to the organization of outpatient GR were lacking practical guidance, lacking adequate exercise and treatment facilities in outpatient GR, staff scarcity, and difficulties arranging staff formation to the varying number of (outpatient) GR trajectories.

The vast majority of care professionals want to increase the application of outpatient GR. They expect an added value for either the patient (e.g., outpatient treatment advantages), GR organization (e.g., more efficient care planning), or society (e.g., expected lower costs). It was frequently indicated that patients recover best in their home environment because this environment offers several treatment advantages, such as the ability to observe the patient in executing activities of daily living and subsequently functionally exercise. Although, at home, there might be a risk that informal caregivers take over many tasks and create dependability. Furthermore, in facility-based outpatient GR, group therapy allows for a more efficient care planning as well as advantages for the patient such as a motivating environment among their peers. In general, some care professionals expect that outpatient GR might reduce costs and pressure on the clinical beds. However, several prerequisites seem necessary to be fulfilled according to the care professionals, such as a suitable reimbursement system and the establishment of care pathways or guidelines for outpatient GR. Furthermore, a positive collaboration of all stakeholders is considered necessary through, for instance, good communication among involved care professionals, positive attitudes of the management and care professionals of GR facilities, and increased involvement of health insurers in, for instance, facilitating eHealth use and manageable information about outpatient GR (e.g., on organization and eligible patients).

The current results seem in line with the European consensus that GR should preferably be offered in an outpatient way [17] and with the study of van den Besselaar and colleagues [22], which indicated that GR providers want an increased utilization of outpatient GR. However, although this study revealed an urge to increase outpatient GR, it also revealed several barriers. One important barrier, the current reimbursement regulations, was also reported in previous studies performed in the Netherlands [18,21,22]. Nonetheless, the present study also indicated that outpatient GR is expected to be cost-saving and contributive to healthcare sustainability, which seems supported by recent, preliminary evidence [19]. Furthermore it was indicated that some patients do not want an outpatient trajectory. However, this is likely to be a limited group of patients because a recent scoping review showed patients experience a need for home-based GR [31]. Furthermore, this study also indicated staff shortage as a barrier. However, a notable addition was that, when using outpatient GR, it is especially complicated to adequately align staff formation to GR demand since the total (outpatient) GR trajectories vary strongly per period.

Furthermore, patients appear to have low activity levels during inpatient rehabilitation [32]. The current study indicated that according to GR professionals, the home environment could stimulate independent activity of the patient. Accordingly, Ramsey and colleagues showed that patients who received home-based GR showed at least a 2.4 times higher daily physical activity compared to hospital-based GR patients for almost all physical activities [33]. This might be a consequence of the “enriched environment” (e.g., environment that invites practicing) that Tijsen and colleagues identified as an aspect of a challenging rehabilitation climate [32]. For instance, the home environment might offer a naturally personalized rehabilitation climate that may invite independent patient activity and practicing. Furthermore, in line with the current study, Tijsen and colleagues indicated that “group therapy” can increase therapy time with the same staff formation [32]. Furthermore, increased therapy time can also be achieved with extra exercising with the informal caregiver [32]. However, the current results also indicate that at home, informal caregivers might take over many tasks of the patient and might therefore create patient dependability. Therefore, informal caregivers should be actively involved in the (outpatient) GR trajectory to potentially improve treatment outcomes in GR patients, such as functional performance and self-efficacy [34].

Although more research is required, studies show that eHealth potentially improves GR outcomes, enhances safety at home [35,36], offers options to create a challenging rehabilitation climate, and is promising for reducing costs and improving patient access to rehabilitation [37,38]. Accordingly, the present study indicated that eHealth should increasingly be used in outpatient GR; however, contextual regulations might hinder this. In the Netherlands, for instance, eHealth requires safety and proven effectiveness before it is included in basic healthcare insurance [39], which financially complicates piloting. Facilitation might be achieved by executing subsidy programs for promising treatments that are not yet reimbursed [39,40]. Regardless, the impact of eHealth in GR might be limited since poor eHealth literacy tends to occur in individuals who are significantly older and affected by multiple chronic conditions [41].

This study has several limitations. First, it is possible that the participants tended to answer in a socially desirable way, which might have decreased the credibility of the data. However, to minimize this potential social desirability bias, the participants were reminded shortly before the interview that their data (including organization names) were going to be processed in an anonymized way in the study report. In addition to this, a member check was performed to improve the data credibility. Second, it is unclear whether the answers provided by the participants in our study adequately reflect the opinion of GR professionals in general. Although we managed to include a variety of care professionals in GR and reached an acceptable level of data saturation, it remains possible that we missed some relevant insights. Moreover, no patients were interviewed and therefore it remains unclear whether expressions made by the care professionals about the patient perspective are trustworthy. A strength of the study is the qualitative approach which allowed for relatively rich information to be obtained in a short period, adding value to the studies that often have a quantitative nature.

It is recommended to use the present findings to develop and evaluate new ways of organizing and reimbursing outpatient GR. These pilot studies should be monitored and evaluated by all important stakeholders including patients and care professionals in both GR and primary care and health insurers. Furthermore, based on these pilots, best practices, and the scientific literature, practice- and evidence-based guidance for outpatient GR should be developed. Subsequently, it should be assessed whether the adjusted outpatient GR is indeed feasible and more cost-effective than inpatient GR.

## 5. Conclusions

GR professionals seem to consider outpatient GR to have added value for patients, GR organizations, and society (e.g., sustainable healthcare). However, various patient-related, care-professional-related, efficiency-related, and organization-related factors seem to negatively influence the current feasibility of outpatient GR (e.g., reimbursement regulations, lack of practical guidance). Despite this, there seems to be agreement on the need to increase the use of outpatient GR. Important experienced prerequisites to facilitate this include incentivizing change in the reimbursement system, development of guidance for outpatient GR, sufficient support for the patients at home, and exploration of the potential role of eHealth in outpatient GR.

## Figures and Tables

**Table 1:** Participating GR disciplines.

GR Disciplines	Number
Physicians specialized in the care for older persons	11
Physiotherapists	5
GR managers *	5
Nurse practitioners	2
Occupational therapists	1

* Executive-level managers (i.e., GR facility-level or GR team-/department-level).

## Data Availability

Data available on request due to privacy restrictions. The coded and summarized data presented in this study are available (in Dutch) on request from the corresponding author. The unprocessed data are not publicly available due to the qualitative nature of the research, which means that in the unprocessed data, a lot of details are included, which cannot be shared because they might be linked to specific persons or organizations.

## References

[1] Kotsani M., Kravvariti E., Avgerinou C., Panagiotakis S., Bograkou Tzanetakou K., Antoniadou E., Karamanof G., Karampeazis A., Koutsouri A., Panagiotopoulou K. (2021). The Relevance and Added Value of Geriatric Medicine (GM): Introducing GM to Non-Geriatricians. J. Clin. Med..

[2] Grund S., Gordon A.L., van Balen R., Bachmann S., Cherubini A., Landi F., Stuck A.E., Becker C., Achterberg W.P., Bauer J.M. (2020). European Consensus on Core Principles and Future Priorities for Geriatric Rehabilitation: Consensus Statement. Eur. Geriatr. Med..

[3] de Groot A.J., Everink I., van Iersel M.B., Smalbrugge M., van de Pol M., Petrovic M., de Rooij S., Rikkert M.G.M.O. (2017). Geriatrische Revalidatiezorg. Leerboek Geriatrie.

[4] Karol R.L. (2014). Team Models in Neurorehabilitation: Structure, Function, and Culture Change. NeuroRehabilitation.

[5] Ellis G., Sevdalis N. (2019). Understanding and Improving Multidisciplinary Team Working in Geriatric Medicine. Age Ageing.

[6] National Health Care Institute (2021). Geriatrische Revalidatiezorg in de Zvw; Artikel 2.5c Bzv: “To Be or Not to Be”?.

[7] Bachmann S., Finger C., Huss A., Egger M., Stuck A.E., Clough-Gorr K.M. (2010). Inpatient Rehabilitation Specifically Designed for Geriatric Patients: Systematic Review and Meta-Analysis of Randomised Controlled Trials. BMJ.

[8] van Oostrom S.H., Picavet H.S.J., de Bruin S.R., Stirbu I., Korevaar J.C., Schellevis F.G., Baan C.A. (2014). Multimorbidity of Chronic Diseases and Health Care Utilization in General Practice. BMC Fam. Pract..

[9] National Institute for Public Health and the Environment Synthese|De Impact van de Vergrijzing. https://www.vtv2018.nl/impact-van-de-vergrijzing.

[10] Nolte E., Knai C., Nolte E., Knai C., Saltman R. (2014). Introduction. Assessing Chronic Disease Management in European Health Systems: Concepts and Approaches.

[11] Van Dam F., De Groot C., Verwest F., Ruimtelijk Planbureau Den Haag (2006). Krimp en Ruimte: Bevolkingsafname, Ruimtelijke Gevolgen en Beleid.

[12] Statistics Netherlands Zal Vergrijzing Leiden tot een Tekort aan Arbeidskrachten?. https://www.cbs.nl/nl-nl/nieuws/2015/20/zal-vergrijzing-leiden-tot-een-tekort-aan-arbeidskrachten-.

[13] Abeliansky A.L., Algur E., Bloom D.E., Prettner K. (2020). The Future of Work: Meeting the Global Challenges of Demographic Change and Automation. Int. Labour Rev..

[14] van Wijk M. Arbeidsmarktprofiel van Zorg en Welzijn. https://www.cbs.nl/nl-nl/longread/statistische-trends/2020/arbeidsmarktprofiel-van-zorg-en-welzijn?onepage=true#c-6-Arbeidsomstandigheden-.

[15] Mirzaie M., Darabi S. (2017). Population Aging in Iran and Rising Health Care Costs. Salmand Iran. J. Ageing.

[16] Kiel S., Zimak C., Chenot J.-F., Schmidt C.O. (2020). Evaluation of an Ambulatory Geriatric Rehabilitation Program—Results of a Matched Cohort Study Based on Claims Data. BMC Geriatr..

[17] van Balen R., Gordon A.L., Schols J.M.G.A., Drewes Y.M., Achterberg W.P. (2019). What Is Geriatric Rehabilitation and How Should It Be Organized? A Delphi Study Aimed at Reaching European Consensus. Eur. Geriatr. Med..

[18] van den Bosch L., Lardinois M., Molenaar N., Schimmel M., Zielman L. (2019). Inventarisatie Zorginhoud GRZ.

[19] Preitschopf A., Holstege M., Ligthart A., Groen W., Burchell G., Pol M., Buurman B. (2023). Effectiveness of Outpatient Geriatric Rehabilitation after Inpatient Geriatric Rehabilitation or Hospitalisation: A Systematic Review and Meta-Analysis. Age Ageing.

[20] de Groot A.J., Vreeburg E.M. (2019). Geriatrische Revalidatiezorg: Samen Werken Aan Herstel!. Bijblijven.

[21] Vreeburg E., de Groot A., Achterberg W. Regeltjes Staan Beste Revalidatiezorg in de Weg. https://www.medischcontact.nl/nieuws/laatste-nieuws/artikel/regeltjes-staan-beste-revalidatiezorg-in-de-weg.htm#:~:text=Maakambulantrevaliderenvoorkwetsbareouderenbetertoegankelijk&text=Geriatrischerevalidatiezorghelptouderenna,regelgevingenf.

[22] van den Besselaar J., Buurman B., Hartel L., Jongenburger A., van Peppen R., Stornebrink E., Wan J. (2021). Varen Op Ervaringen.

[23] Kroes J., van Buul L., Veenhuizen R., de Groot A., Depla M., Vergeer W., Hertogh C. (2018). Ambulante Geriatrische Zorg Voor Kwetsbare Thuiswonende Ouderen: Een Haalbaarheidsonderzoek. Tijdschr. Gerontol. Geriatr..

[24] Dutch Healthcare Authority Regeling Medisch-Specialistische Zorg—NR/REG-2207a. https://puc.overheid.nl/nza/doc/PUC_720738_22/.

[25] de Groot A.J., de Vries-Lim I., Trumpi A.M.A., Hertogh C.M.P.M., Van der Wouden J.C. Ambulante Geriatrische Revalidatie Bij Ziekte van Parkinson. https://www.verenso.nl/magazine-februari-2015/no-1-februari-2015/praktijk/ambulante-geriatrische-revalidatie.

[26] Polit D., Beck C., Polit D., Beck C. (2019). Qualitative Research Design and Approaches. Nursing Research.

[27] Saunders B., Sim J., Kingstone T., Baker S., Waterfield J., Bartlam B., Burroughs H., Jinks C. (2018). Saturation in Qualitative Research: Exploring Its Conceptualization and Operationalization. Qual. Quant..

[28] Polit D., Beck C., Polit D., Beck C. (2019). Sampling in Qualitative Research. Nursing Research.

[29] Polit D., Beck C., Polit D., Beck C. (2019). Qualitative Data Analysis. Nursing Research.

[30] Polit D., Beck C., Polit D., Beck C. (2019). Trustworthiness and Rigor in Qualitative Research. Nursing Research.

[31] Lubbe A.L., van Rijn M., Groen W.G., Hilhorst S., Burchell G.L., Hertogh C.M.P.M., Pol M.C. (2023). The Quality of Geriatric Rehabilitation from the Patients’ Perspective: A Scoping Review. Age Ageing.

[32] Tijsen L., Derksen E., Achterberg W., Buijck B.I. (2019). Challenging Rehabilitation Environment for Older Patients. Clin. Interv. Aging.

[33] Ramsey K.A., Loveland P., Rojer A.G.M., Denehy L., Goonan R., Marston C., Kay J.E., Brenan J., Trappenburg M.C., Lim W.K. (2021). Geriatric Rehabilitation Inpatients Roam at Home! A Matched Cohort Study of Objectively Measured Physical Activity and Sedentary Behavior in Home-Based and Hospital-Based Settings. J. Am. Med. Dir. Assoc..

[34] Blanton S., Scheibe D.C., Rutledge A.H., Regan B., O’Sullivan C.S., Clark P.C. (2019). Family-Centered Care During Constraint-Induced Therapy After Chronic Stroke: A Feasibility Study. Rehabil. Nurs..

[35] Bernocchi P., Giordano A., Pintavalle G., Galli T., Ballini Spoglia E., Baratti D., Scalvini S. (2019). Feasibility and Clinical Efficacy of a Multidisciplinary Home-Telehealth Program to Prevent Falls in Older Adults: A Randomized Controlled Trial. J. Am. Med. Dir. Assoc..

[36] Kraaijkamp J.J.M., van Dam van Isselt E.F., Persoon A., Versluis A., Chavannes N.H., Achterberg W.P. (2021). EHealth in Geriatric Rehabilitation: Systematic Review of Effectiveness, Feasibility, and Usability. J. Med. Internet Res..

[37] Bakhshayesh S., Hoseini B., Bergquist R., Nabovati E., Gholoobi A., Mohammad-Ebrahimi S., Eslami S. (2020). Cost-Utility Analysis of Home-Based Cardiac Rehabilitation as Compared to Usual Post-Discharge Care: Systematic Review and Meta-Analysis of Randomized Controlled Trials. Expert Rev. Cardiovasc. Ther..

[38] Kraaijkamp J.J.M., Van Dam Van Isselt E., Persoon A., Chavannes N., Achterberg W. EHealth in de Geriatrische Revalidatie: Lessen Uit Een Systematic Review over Effectiviteit, Haalbaarheid En Bruikbaarheid. https://www.verenso.nl/magazine-augustus-2020/no-4-augustus-2020/wetenschap/ehealth-in-de-geriatrische-revalidatie.

[39] Dutch Healthcare Authority Wegwijzer Bekostiging Digitale Zorg 2023. https://puc.overheid.nl/nza/doc/PUC_655318_22/1/.

[40] Bruins B., Ministry of Health Welfare and Sport Kamerbrief over Aanbieding EHealth-Monitor en Stand van Zaken Slimme Zorg. https://www.rijksoverheid.nl/onderwerpen/e-health/documenten/kamerstukken/2019/11/01/kamerbrief-over-aanbieding-e-healthmonitor-en-stand-van-zaken-slimme-zorg.

[41] Smith B., Magnani J.W. (2019). New Technologies, New Disparities: The Intersection of Electronic Health and Digital Health Literacy. Int. J. Cardiol..

